# Monoclonal Antibodies against Nucleocapsid Protein of SARS-CoV-2 Variants for Detection of COVID-19

**DOI:** 10.3390/ijms222212412

**Published:** 2021-11-17

**Authors:** Ruei-Min Lu, Shih-Han Ko, Wan-Yu Chen, Yu-Ling Chang, Hsiu-Ting Lin, Han-Chung Wu

**Affiliations:** 1 Biomedical Translation Research Center (BioTReC), Academia Sinica, Taipei 11529, Taiwan; reminlu@gate.sinica.edu.tw (R.-M.L.); shko@gate.sinica.edu.tw (S.-H.K.); a29259963@hotmail.com (Y.-L.C.); 2 Institute of Cellular and Organismic Biology, Academia Sinica, Taipei 11529, Taiwan; sirirem59@gmail.com (W.-Y.C.); 3772.monico@gmail.com (H.-T.L.)

**Keywords:** COVID-19, SARS-CoV-2, antibody, nucleocapsid protein, rapid test, LFIA

## Abstract

Mitigation strategies of the coronavirus disease 2019 (COVID-19) pandemic have been greatly hindered by the continuous emergence of SARS-CoV-2 variants. New sensitive, rapid diagnostic tests for the wide-spectrum detection of viral variants are needed. We generated a panel of 41 monoclonal antibodies against the SARS-CoV-2 nucleocapsid protein (NP) by using mice hybridoma techniques. Of these mAbs, nine exhibited high binding activities and were applied in latex-based lateral flow immunoassays (LFIAs). The LFIAs utilizing NP-mAb-7 and -40 had the best sensitivity and lowest limit of detection: 8 pg for purified NP and 625 TCID_50_/mL for the authentic virus (hCoV-19/Taiwan/4/2020). The specificity tests showed that the NP-mAb-40/7 LFIA strips did not cross-react with five human coronavirus strains or 20 other common respiratory pathogens. Importantly, we found that 10 NP mutants, including alpha (B.1.1.7), beta (B.1.351), gamma (P.1), and delta (B.1.617.2) variants, could be detected by NP-mAb-40/7 LFIA strips. A clinical study (*n* = 60) of the NP-mAb-40/7 LFIA strips demonstrated a specificity of 100% and sensitivity of 90% in infected individuals with cycle threshold (Ct) values < 29.5. These anti-NP mAbs have strong potential for use in the clinical detection of SARS-CoV-2 infection, whether the virus is wild-type or a variant of concern.

## 1. Introduction

Coronavirus disease 2019 (COVID-19) is caused by severe acute respiratory syndrome coronavirus 2 (SARS-CoV-2) infection and has become a global public health crisis in a remarkably short time [1]. According to the World Health Organization (WHO), there have been over 200 million confirmed cases of COVID-19 and 4.4 million deaths worldwide as of August 2021. The upper respiratory tract and lungs are the organs most affected by COVID-19, because the virus enters host cells via an interaction between the viral spike protein and the human receptor angiotensin-converting enzyme 2 (ACE2), which is most abundant on the surface of type II alveolar cells of the lungs [2]. Although 80% of infections are not severe and the patients recover without medical intervention, 15% of individuals with confirmed infection require hospitalization and 5% develop severe illness [3]. The overall mortality risk is 0.5–1.0%, but the risk is much higher in the elderly (>85 years, 10–27% mortality) and those with other risk factors [3]. Despite remarkable recent successes in the development of vaccines and therapeutic antibodies, the COVID-19 case numbers continue to increase, and the disease still poses a serious threat to healthcare systems in most countries [4,5].

SARS-CoV-2 belongs to the family *Coronaviridae* and is a positive-sense, single-stranded, enveloped RNA virus. The 30-kb genome encodes four essential structural proteins, including spike (S), envelope (E), membrane (M), and nucleocapsid (NP), as well as several nonstructural proteins [6]. NP is a multifunctional RNA-binding protein that plays many crucial roles in the packaging of the viral RNA genome, regulating viral RNA synthesis during replication and transcription and facilitating virus particle assembly [7]. NP consists of five domains: the *N*-terminal tail region (NTD), RNA-binding domain (RBD), a Ser/Arg-rich linker region (SR-rich region), a dimerization domain (DD), and the *C*-terminal intrinsically disordered domain (CTD) [8]. Among coronavirus-produced proteins, NP is the most abundant, and it elicits high titers of binding antibodies during humoral immune responses. In addition, NP functions to accelerate the viral life cycle by modulating the host cell immune response. Most recently, Ma et al. showed that NP binds the Gasdermin D linker region and hinders Gasdermin D cleavage by caspase-1 in monocytes, leading to the inhibition of host cell pyroptosis [9]. Kang et al. isolated a high-affinity anti-NP mAb from a convalescent COVID-19 patient and found that the binding of the mAb to the RBD of NP compromised the induction of complement hyperactivation by NP, a risk factor for the morbidity and mortality of COVID-19 patients [10]. Interestingly, viral NP and the RNA genome are detectable in blood specimens of SARS-CoV-2-infected patients, and the levels correlate with mortality in COVID-19 ICU patients [11,12]. Therefore, the generation of anti-NP antibodies can be used to reveal viral NP functions in viral pathogenesis, and more importantly, the anti-NP antibodies may be useful when applied in diagnostic assays for the detection of SARS-CoV-2 infection, such as serologic and antigen tests [13,14,15].

Sensitive, specific, and rapid diagnostic tests for SARS-CoV-2 infection are vital for maintaining the public health, especially with regards to identifying asymptomatic but infectious individuals [16,17]. Like the tests for other respiratory viruses, both viral nucleic acids and antigens can be detected from nasopharyngeal swab specimens, and these methods have been used extensively for the screening and early diagnosis of SARS-CoV-2 infection [15]. Among the available methods, real-time (quantitative) reverse transcription polymerase chain reaction (RT-PCR) is the current gold standard for a COVID-19 diagnosis, as it can be used to detect low levels of SARS-CoV-2 nucleic acids and shows a high analytical accuracy. The typical RT-PCR methods for detecting the virus require four to six hours from the acquisition of a clinical sample to the results, and the test must be performed in certificated laboratories with well-trained technicians and expensive equipment [18]. On the other hand, antigen tests based on lateral flow immunoassay (LFIA) technology are comparatively less expensive and do not require specialized laboratory skills. Antigen testing also offers quick turnaround times, as results may be available within 15 min. Based on these advantages, antigen tests are thought to be well-suited for the large-scale detection of COVID-19 among high-risk groups and in high-density environments; such point-of-care tests can provide simple, rapid, and cost-effective assays [19].

The widespread availability of accurate and efficient diagnostic testing is a key component of effective strategies to combat the COVID-19 pandemic, and efforts to develop molecular and antigen tests for the diagnosis of COVID-19 have already been successful. However, the SARS-CoV-2 virus has mutated over time, resulting in genetic variants that now dominate the circulating viral strains. Since most diagnostic tests were designed to detect the original virus, the emergence of new SARS-CoV-2 variants may negatively impact the sensitivity of COVID-19 tests by potentially increasing the false-negative rates [20]. Mutations in the S protein, especially those in the viral variants of concern (VoCs), have attracted much recent attention, because immune escape by mutants could compromise the neutralizing functions of therapeutic antibodies and antibodies present in vaccinated individuals [21]. Although NP is thought to be more conserved and stable than the S protein, dozens of mutations in NP have been found in the emerging viral variants [22,23]. Until now, there have been very few reports in which the clinical performance of LFIA antigen tests was validated with respect to the SARS-CoV-2 variants [24,25]. Hence, the development of diagnosis kits that ensure the comprehensive detection of the mutant viral antigens in the common variants is urgently needed.

Herein, we generated novel mAbs against SARS-CoV-2 NP and constructed highly sensitive LFIA strips. We further validated the performance of the antigen strip in clinical samples. Overall, this report describes the bench-to-bedside development of mAb-40/7 LFIA strips, which have been granted Emergency Use Authorization (EUA) by the Taiwan Food and Drug Administration (TFDA).

## 2. Results

### 2.1. Generation and Identification of mAbs against SARS-CoV-2 NP

**Figure 1:**
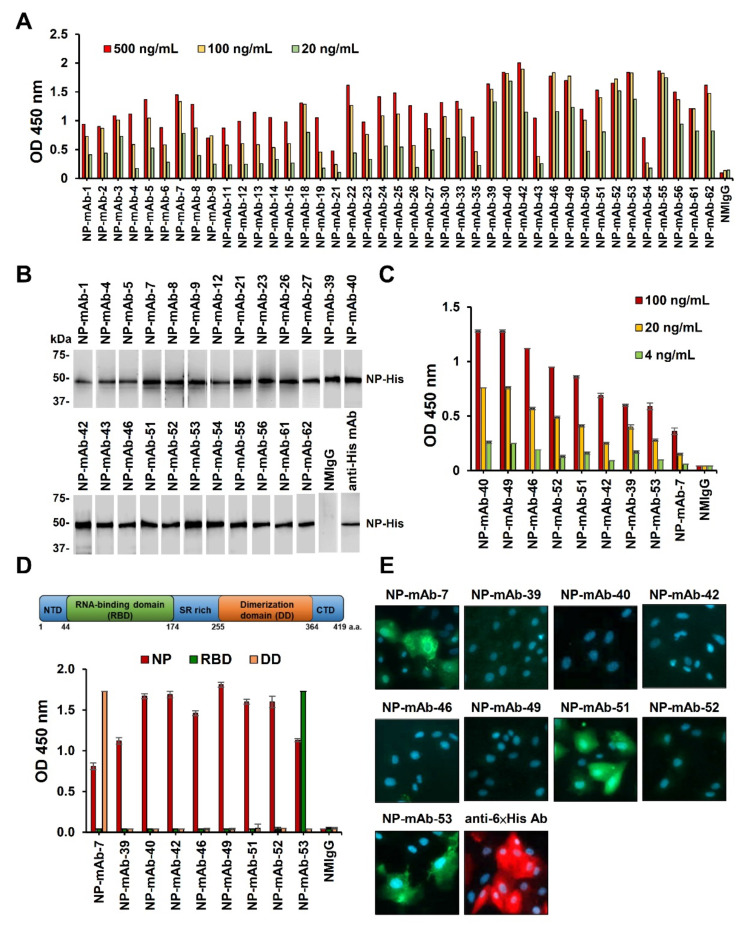
Generation of mAbs against SARS-CoV-2 NP. (**A**) Binding activity of 41 anti-NP mAbs was performed by ELISAs that were coated in recombinant NP-His protein (2 μg/mL). Each anti-NP mAb was serially diluted from 500 to 20 ng/mL. (**B**) Thirty-four anti-NP mAbs were used as the primary antibody for NP-His immunoblots. Anti-6 × His mAb was used as a positive control. (**C**) Comparative ELISAs were performed to evaluate the binding activities of mAbs against NP-His. Recombinant NP-His (0.5 μg/mL) was coated on the ELISA plates. Normal mouse IgG (NMIgG) was used as a negative control. (**D**) The binding activity of nine mAbs against recombinant full-length NP, the RNA-binding domain (RBD) of NP, and dimerization domain (DD) of NP by ELISA. (**E**) NP-expressing Ver-E6 cells were probed with 1 μg/mL anti-NP mAbs and then stained with FITC goat anti-mouse IgG. NP expression was confirmed by staining with anti-6 × His Ab and rhodamine fluorescence detection. Abbreviation: NTD, *N*-terminal domain; SR rich, Serine/Arginine-rich region; CTD, *C*-terminal domain.

To generate novel mAbs that specifically bind to SARS-CoV-2 NP, we immunized BALB/c mice with recombinant NP-His. Forty-one mAbs against NP were generated using the mouse hybridoma technique, with the mAb reactivity measured by ELISA (Figure 1A). Among the antibody clones, 24 could recognize recombinant NP at its expected molecular weight of ~50 kDa in a Western blot analysis (Figure 1B). The other 17 mAbs did not react with the linear epitopes. Using ELISA, we identified nine mAbs with strong binding activity against NP, i.e., NP-mAb-40, -49, -46, -52, -51, -42, -39, -53, and -7 (Figure 1C). The hybridoma cells of these nine mAbs were separately expanded in ascites, and the purified antibodies were confirmed by SDS-PAGE (Supplementary Figure S1A). The antibody isotypes of the nine mAbs belonged to the IgG1/kappa class (Supplementary Figure S1B). NP is composed of three main parts: the *N*-terminal RNA-binding domain (RBD), an SR-rich linker, and the *C*-terminal dimerization domain (DD) [7]. To determine the binding domains for the nine mAbs, we performed ELISA using RBD and DD recombinant proteins (Figure 1D). NP-mAb-7 and -53 bound to RBD and DD, respectively. The binding epitopes of the other seven mAbs appeared to be outside of these two domains, since they had no positive signals in the ELISAs using RBD and DD. We further showed that NP-mAb-7, -51, and -53 can stain NP-expressing Vero E6 cells but not mock-transfected cells, indicating that these three mAbs can be used for immunofluorescence assay (IFA) experiments (Figure 1E and Supplementary Figure S1C). The identification and characterization of the 41 mAbs are summarized in Table 1. Due to their high activities and specificities, the nine mAbs with strong binding were further evaluated for use in lateral flow immunoassays (LFIAs) to detect SARS-CoV-2 NP.

### 2.2. Screening of Antibody Pairs in Lateral Flow Immunoassays (LFIAs) to Detect NP Antigen

**Figure 2:**
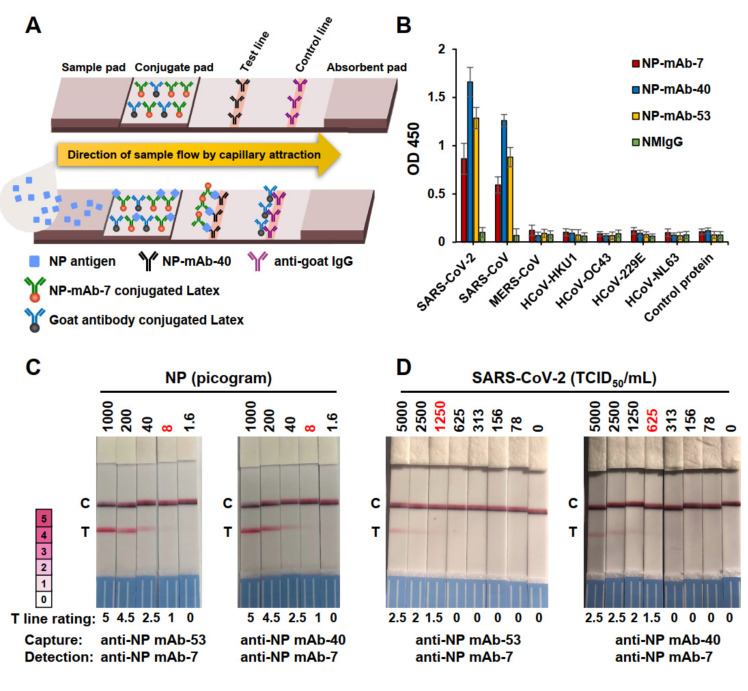
Detection limit of two LFIAs using different capture mAbs. (**A**) Schematic diagram of the lateral flow rapid test configuration. (**B**) Binding activities of anti-NP mAb-7, -40, and -53 to NPs from seven types of human coronaviruses (HCoV). Each recombinant NP-His was coated at the same concentration on the ELISA plates. Anti-NP mAbs were added at 100 ng/mL to the probe antigens. (**C**) SARS-CoV-2-infected Vero E6 cells were collected in a lysis buffer, and the NP concentration was determined by sandwich ELISA. The samples were diluted to given concentrations and analyzed by two antigen rapid tests with different capture mAbs. The rating chart showed an intensity range from 0 to 5 grading of the positive and negative results on the test line. A color intensity ≥ 0.5 was judged as a positive result. A color intensity < 0.5 was judged as a negative result. (**D**) Authentic SARS-CoV-2 virus concentrations ranging from 0 to 5000 TCID_50_/mL were used to detect the sensitivities of two antigen rapid tests. The numbers in red indicate the limits of detection (LODs).

Constructing an LFIA strip involves fixing a detection antibody on the test line of the strip and coupling it with a capture antibody that is conjugated to latex nanoparticles. The design and principle of antigen detection using an LFIA strip are illustrated in Figure 2A. The nine mAbs with strong binding to NP were tested as capture or detection antibodies, constituting a total of 81 antibody pairings in the candidate lateral flow assays (Supplementary Table S1). We screened the 81 prototype assays for NP detection and found that most did not yield positive signals when NP was applied. However, the antibody pairings of NP-mAb-40/7, -39/53, -52/53, and -53/7 were highly sensitive for the detection of NP generated from *Escherichia coli* and 293T cells; the signal intensity values for these pairings were all ≥2.5 (Supplementary Table S1). When detecting NP from 293T cells, the binding signals of NP-mAb-40/7 and -53/7 were higher than those of mAb-39/53 and -52/53. Thus, we chose to focus on NP-mAb-40/7 and -53/7 for our further studies. To evaluate the specificity of NP-mAb-7, -40, and -53 for SARS-CoV-2 NP, we performed ELISAs to determine the binding to the NPs of non-SARS-CoV-2 human coronavirus strains, including two alpha coronaviruses (HCoV-229E and HCoV-NL63), two beta coronaviruses (HCoV-HKU1 and HCoV-OC43), and two severely pathogenic forms (MERS-CoV and SARS-CoV) (Figure 2B). The amino acids sequences of the NPs from SARS-CoV-2 and SARS-CoV were up to 93.6% similar and 90.0% identical, so it was not surprising that NP-mAb-7, -40, and -53 cross-reacted with the NP of SARS-CoV but not the other five virus strains.

Next, we analyzed the lowest detectable concentrations (limits of detection; LODs) for the NP-mAb-40/7 and -53/7 LFIA strips when detecting viral NP in the cell lysates of SARS-CoV-2-infected Vero E6 cells (Figure 2C). Both LFIA strips showed identical LODs of 8-pg viral NP. The performances of the NP-mAb-40/7 and -53/7 LFIA strips were then further evaluated against the authentic virus (Figure 2D). Serial dilutions of gamma-irradiated SARS-CoV-2 (hCoV-19/Taiwan/4/2020) were added to the strips. We found that NP-mAb-40/7 LFIA strips were more sensitive than NP-mAb-53/7 LFIA strips for the detection of an inactivated virus, with an LOD of 625 TCID_50_/mL for the NP-mAb-40/7 LFIA and 1250 TCID_50_/mL for the NP-mAb-53/7 LFIA. Thus, we chose NP-mAb-40/7 LFIA strips for the preclinical studies. To determine a more precise LOD in a clinically relevant sample, we spiked serial dilutions of the inactivated virus (USA-WA1/2020) into a pooled human nasal matrix (Clinical Nasal Matrix, CNM) and measured the LOD (Table 2). A preliminary LOD was measured with a cutoff of a ≥95% positive rate in nine replicates at each concentration. We found that the samples with 547 TCID_50_/mL had a 100% positive rate, whereas 273 TCID_50_/mL had an 88.9% positive rate. Next, we performed 20 additional replicates using the NP-mAb-40/7 LFIA to detect the apparent LOD concentration of 547 TCID_50_/mL (Table 2). Based on the 100% positive rate, we concluded that the LOD of the NP-mAb-40/7 LFIA strips was indeed 547 TCID_50_/mL.

### 2.3. Performance of NP-mAb-40/7 LFIA Strips for NP of SARS-CoV-2 Variants

**Figure 3:**
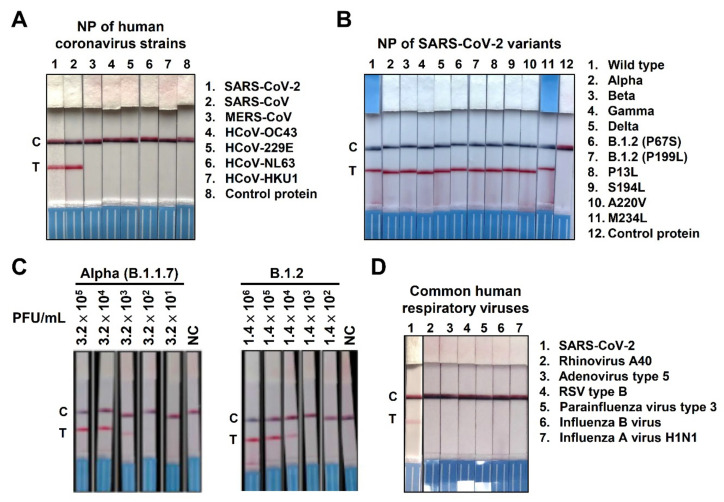
Specificity of NP mAb-40/7 LFIA strips. (**A**) Detection of viral NP of human coronavirus strains was performed by loading 10 ng of the indicated recombinant protein onto NP mAb-40/7 LFIA strips, followed by a 10-min reaction time. (**B**) The indicated recombinant SARS-CoV-2 NP variants were loaded onto NP mAb-40/7 LFIA strips at 1 ng. Mutations of D3L, R203K, G204R, and S235F have been identified in lineage B.1.1.7 (lane 2). A mutation of T205I was reported in lineage B.1.351 (lane 3); P80R was identified in lineage P.1 (lane 4); and D63G, R203M, D377Y, and R385K were found in lineage B.1.617.2 (lane 5). NC indicates the negative control. (**C**) NP mAb-40/7 LFIA strips were used to detect clinically identified alpha variant lineage B.1.1.7 and epsilon variant B.1.2 by loading the indicated viral titer (PFU/mL). NC indicates the negative control. (**D**) Several respiratory associated viruses were not detected by NP mAb-40/7 LFIA strips. SARS-CoV-2 served as a positive control (lane 1). The detecting concentration of the indicated viruses was 10^5^ copies/reaction of SARS-CoV-2, adenovirus type 5, rhinoviruses A40, respiratory syncytial virus type B1, parainfluenza virus type 3, and influenza A virus H1N1. Influenza B virus utilized 10^6^ copies/reaction for detection.

To test the specificity of the NP-mAb-40/7 LFIA strips, we loaded recombinant NP from seven different human coronaviruses on the strips (Figure 3A). The NP-mAb-40/7 LFIA strips specifically recognized SARS-CoV-2 NP, only cross-reacting with SARS-CoV NP. MERS-CoV and endemic coronavirus NP were not detectable, even when loading high amounts of recombinant NP (10 ng). These data corresponded with our ELISA results (Figure 2B). Since several SARS-CoV-2 variants have emerged and circulated around the world during the COVID-19 pandemic, a number of nonsynonymous mutations in NP have been identified through sequence-based surveillance [23]. For example, the D3L, R203K, G204R, and S235F mutations in NP were reported in the alpha variant (B.1.1.7), while the D63G, R203M, D377Y, and R385K mutations in NP were found in the delta variant (B.1.617.2). To test whether mutations in these variants might lead to false-negative results with the NP-mAb-40/7 LFIA strips, we analyzed recombinant NPs from four variants of concern (VoCs), as well as an NP harboring high-frequency mutations (Figure 3B). The strips showed positive signals when detecting all ten mutant NPs, indicating that most virus variants can be detected by these strips. The results also suggested that the tested mutations do not prevent NP-mAb-7 and -40 from binding to their epitopes. Furthermore, we examined whether NP-mAb-40/7 LFIA strips can recognize the circulating SARS-CoV-2 variants, alpha (B.1.1.7) and B.1.2 (Figure 3C). The alpha and B.1.2 variants are, respectively, estimated to have a cumulative prevalence of 25% and 2% worldwide [26]. The alpha variant carries E474K and L452R mutations in the spike protein, as well as D3L, R203K, S235F, and G204R in NP [27]. Lineage B.1.2 contains a Q667P mutation in the spike protein and P199L and P67S mutations in NP [28]. Both authentic virus variants could be recognized by NP-mAb-40/7 LFIA strips (Figure 3C).

### 2.4. Specificity of NP-mAb-40/7 LFIA Strips

We evaluated the potential cross-reactivity of NP-mAb-40/7 LFIA strips with common pathogens that may cause human upper respiratory diseases (Figure 3D). Six virus strains, including adenovirus, rhinovirus, respiratory syncytial virus (RSV), parainfluenza virus, influenza virus A, and influenza virus B, yielded negative results when applied to NP-mAb-40/7 LFIA strips. We then tested 10 bacterial and 12 virus strains spiked into the human nasal matrix; none of the pathogenic agents were recognized by NP-mAb-40/7 LFIA strips, nor did any of the agents interfere with the detection of SARS-CoV-2 (Supplementary Table S2). In addition, we conducted interference assays on 15 substances commonly found in the human upper respiratory tract of patients seeking medical care, including chemicals, drugs, and biological components. None of these substances measurably influenced the detection of SARS-CoV-2 (Supplementary Table S3). These data suggested that the NP-mAb-40/7 LFIA strips are highly specific to the NP of SARS-CoV-2, even in the presence of a clinical human nasal matrix with abundant respiratory pathogens or other potential interfering substances. Furthermore, we performed accelerated stability testing on the NP-mAb-40/7 LFIA strips. The test strips exhibited triplicate passes of the QC panel over 14 days at 60 °C, which suggests a product shelf-life of 12 months according to the Arrhenius reaction conversion [29].

### 2.5. Clinical Studies of NP-mAb-40/7 LFIA Strips

**Figure 4:**
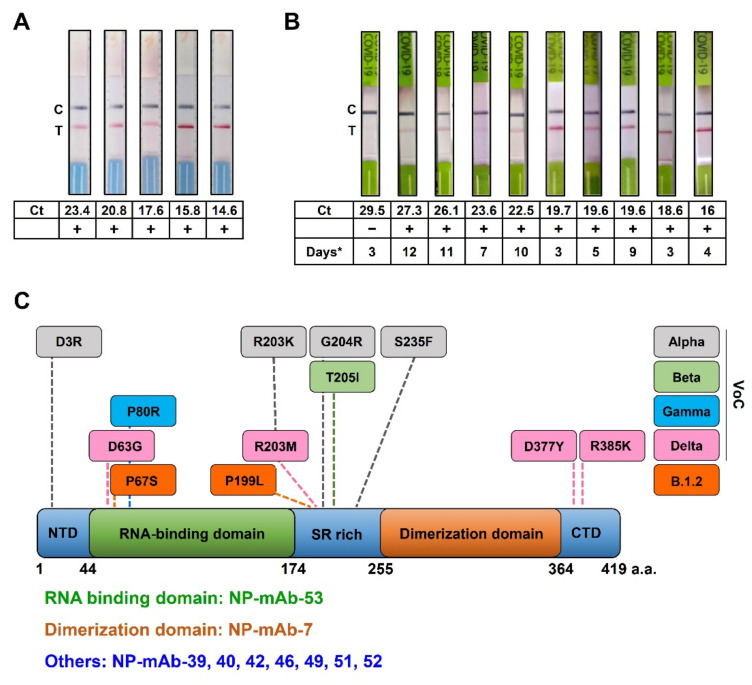
Clinical verification of the antigen rapid tests. (**A**) NP mAb-40/7 LFIA strips were used to probe five frozen samples with Ct values between 14.6 and 23.4 for the viral E gene. (**B**) NP mAb-40/7 LFIA strips were used to probe ten freshly collected positive patient samples with Ct values between 16 and 29.5 for the viral E gene. Days* means the days after onset to sampling. (**C**) Recognition sites of anti-NP mAbs on SARS-CoV-2 NP. SARS-CoV-2 NP contains 419 amino acid residues (a.a.). Each domain length is shown underneath the illustration. Amino acid mutations in SARS-CoV-2 NP are labeled for the different variants of concern (VoCs). The binding domains of the anti-NP mAb we studied are listed in the low panel.

To investigate the clinical utility of NP-mAb-40/7 LFIA strips for SARS-CoV-2 detection, we tested the frozen and fresh nasopharyngeal swabs from COVID-19 patients. First, we examined the frozen samples using NP-mAb-40/7 LFIA strips. The frozen samples (*n* = 5) were validated by RT-PCR as positive for SARS-CoV-2 infection. The NP-mAb-40/7 LFIA strips had a 100% positive rate for the patient frozen samples, which had viral loads of SARS-CoV-2 between 14.6 and 23.4 Ct values, as measured by RT-PCR (Figure 4A). Next, we performed the clinical trial to collect the fresh samples, which was an open-label, nonrandomized study with the planned enrollment of 60 subjects over 20 years old. The inclusion criteria required at least one of the following clinical and epidemiological conditions: fever and other respiratory symptoms and pneumonia confirmed by clinical, radiological, and pathological examination. The epidemiological conditions involved the people who had a contact history with COVID-19 patients (Table 3). We analyzed fresh nasopharyngeal swab specimens from 60 individuals by using NP-mAb-40/7 LFIA strips, and the results of ten RT-PCR-positive samples are shown in Figure 4B. Compared to a standard RT-PCR assay using a Ct cutoff value of less than 30 cycles for positive cases, the NP-mAb-40/7 LFIA strips exhibited a 98.3% overall accuracy, 100% specificity, and 90% sensitivity (95% CI, 55.5–99.8) (Table 4). We also compared the results of the NP-mAb-40/7 LFIA strips with patient clinical outcomes. For the RT-PCR-positive patients, the days of onset to sampling ranged from 3 to 12 days (Figure 4B). We found that early symptomatic COVID-19 cases (up to 5–7 days after symptom onset) [30] with Ct ≤ 27.3 could be detected by the NP-mAb-40/7 LFIA strips. These data suggest that NP-mAb-40/7 LFIA strips display an appropriate diagnostic accuracy for SARS-CoV-2 detection.

## 3. Discussion

It is unclear how long the COVID-19 pandemic will last. Although safe and effective vaccines have been developed, the majority of people in the world have yet to be fully vaccinated. People infected by SARS-CoV-2 continue to suffer from a high risk of hospitalization and severe complications. One component of an effective mitigation strategy is mass and rapid screening to identify virus carriers. The use of RT-PCR to amplify viral nucleic acids has shown excellent specificity and sensitivity and become the gold standard for the laboratory diagnosis of COVID-19 [15,31]. However, this technique requires an appropriate laboratory facility and experienced staff, making the method less available to many healthcare facilities, especially those in low- and middle-income countries. For this reason, less expensive, portable, easy, and fast screening tools with reliable results, such as antibody-based LFIA strips, are worthy of development [32].

The diagnostic sensitivity of LFIA is highly correlated with the viral load [33]. In symptomatic patients with SARS-CoV-2 infection, the NP expression levels peak at about 8–14 days after the onset of symptoms [34]. Several COVID-19 antigen detection kits are commercially available and have established benchmarks for analytical and diagnostic performances based on their large-scale use around the world [35]. For example, the clinical performance of the Roche SARS-CoV-2 rapid antigen test was evaluated in hospitals in Italy using nasopharyngeal swab samples from 321 patients, showing a range of sensitivity between 97 and 100% for patients with Ct values <25 and between 50 and 81% for those with Ct values between 25 and <30 [36]. In another example, the Abbott BinaxNOW COVID-19 antigen card was used to screen nasal swab specimens from 2645 asymptomatic individuals, with an analytical sensitivity of 95.8% in patients with Ct values < 23 and 66.7% in those with Ct values < 30; positive results were found in 0% of the samples with Ct values > 30 [37]. The STANDARD Q COVID-19 (SD-Biosensor) was evaluated in a symptomatic and non-hospitalized population of 3615 subjects, showing an overall sensitivity and specificity of 84.9% and 99.5%, respectively. The sensitivity was 99.1% for samples with Ct values ≤ 25 and 94.3% with Ct ≤ 30 [33]. In this study, we found a sensitivity for NP-mAb-40/7 LFIA strips of 90% in patients with Ct values < 30 and 100% with Ct values ≤ 27.3 of symptom onset (Figure 4B). We compared the LOD and clinical performance of the NP-mAb-40/7 LFIA strips with those of commercial NP antigen tests (Supplementary Table S4), finding that our strip was comparable to well-known commercialized COVID-19 antigen rapid tests. Nevertheless, this diagnostic test needs to be further evaluated with expanded sample sizes to validate its efficacy, and the current data support further studies to perform prospective clinical validations in asymptomatic, pre-symptomatic, and symptomatic individuals.

SARS-CoV-2 is a single-stranded RNA virus that has higher mutation rates than DNA viruses [38]. Based on this genomic character, several SARS-CoV-2 VoCs have emerged and become widely distributed during the COVID-19 pandemic. The alpha variant (B.1.1.7) first emerged in the United Kingdom, the beta variant (B.1.351) in South Africa, the gamma variants (P.1) in Brazil, and the delta variant (B.1.617.2) in India. At least some of these variants appear to have enhanced transmissibility and have rapidly become dominant in many populations [39]. In addition to mutations in the spike protein, the NP in these variants are mutated as well (Figure 4C). Such changes bring concerns about whether the mutants retain epitopes for anti-NP mAbs. If not, the binding ability of antibodies targeting NP may be lost, causing false-negative results in NP antigen detection kits. We were therefore careful to design the NP-mAb-40/7 LFIA strips so that they would successfully recognize at least 10 recombinant NP mutants and two authentic virus variants, including alpha (B.1.1.7) and B.1.2 (Figure 3B). These results indicate that the strips may maintain high sensitivities, even when used in areas with the widespread transmission of SARS-CoV-2 variants. In the future, it will be necessary to assess whether NP-mAb-40/7 LFIA strips can detect other authentic virus variants, such as gamma and delta.

According to the current WHO classifications, two strains are designated as Variants of Interest (VOI): Lambda (C.37) and Mu (B.1.621). The database outbreak.info [26] showed that Lambda has P13L, R203K, G204R, and G214C mutations in the NP, and Mu only has a T205I mutation in the NP. We found that three out of four mutants in Lambda, i.e., P13L, R203K, and G204R, could be recognized by NP mAb-40/7 LFIA strips (lanes 2 and 8 in Figure 3B). The T205I mutation in Mu is identical to that in Beta (B.1.351), which could be detected by our strips (lane 3 in Figure 3B). Therefore, these two VOIs have a high probability of being detected by NP-mAb-40/7 LFIA strips. There are also fourteen variants classified as Variants Under Monitoring (VUMs). Of note, Kappa (B.1.617.1), Iota (B.1.526), Eta (B.1.525), and Epsilon (B.1.427/B.1.429) are former VOIs currently designated as VUMs. Kappa has R203M and D377Y mutations, both of which are included in the Delta (B.1.617.2) NP (lane 5, Figure 3B). No mutation was reported in the NP of Iota. Only one mutation, T205I, was found in the NP of Epsilon, and it is identical to that in Beta (lane 3, Figure 3B). Hence, NP-mAb-40/7 LFIA strips might be able to detect Kappa, Iota, and Epsilon. However, Eta harbors S2Y, A12G, and T205Y mutations in the NP, which have been not assessed in our study. In the future, we will thoroughly evaluate the performance of NP-mAb-40/7 LFIA strips for Eta and other VUMs.

We utilized wild-type and truncated recombinant NP proteins, either the RBD (44–174 amino acids) or dimerization domain (255–364 amino acids), to locate the binding regions of our nine candidate anti-NP mAbs. Among the tested NP mAbs, NP-mAb-53 bound the RBD of NP, which is responsible for RNA binding [40]. NP-mAb-7 targeted the dimerization domain of NP, which functions in self-association and virion assembly [7,41] and is likely conserved; few mutations in the dimerization domain have been identified in the SARS-CoV-2 variants (Figure 4C). The binding epitopes of the other seven anti-NP mAbs are probably located in the NTD, SR-rich region, or CTD of NP. These three domains are classified as disordered regions of NP [7]. Interestingly, previous studies have shown that the disorder region is a higher efficient epitope in the process of generate antibodies than the order region [42]. This may explain why most of our anti-NP mAbs bound to the disordered regions. Although the precise epitopes of the anti-NP mAbs will need to be further clarified, the NP mAbs targeting different regions have the potential to become useful tools for studying the functions of different NP domains during viral pathogenesis.

Consequently, NP-mAb-7 and -40 were generated and applied in LFIA strips for the detection of SARS-CoV-2 with good accuracy and stability. NP-mAb-40/7 LFIA strips received an EUA from the TFDA on 8 July 2021 for the diagnosis of COVID-19. The tests have entered the market under the brand name “Acadecise”. We hope that the high-quality mAbs and Acadecise can be useful in helping to curb the circulation of SARS-CoV-2.

## 4. Materials and Methods

### 4.1. Recombinant Proteins and Reagents

For the generation of wild-type recombinant SARS-CoV-2 NP tagged with six histidine residues (NP-His), we cloned cDNA-coding full-length NP into pET21a plasmid DNA (Merck, Darmstadt, Germany) in-frame with a *C*-terminal His-Tag sequence. Wild-type recombinant NP-His were generated in *E.coli* BL21(DE3) and purified by a Ni Sepharose 6 Fast Flow column (Merck, Darmstadt, Germany) according to the manufacturer’s instructions. The recombinant NP of SARS-CoV-2 variants and other coronaviruses was obtained from Sino Biological Inc. (Beijing, China) and ACRO Biosystems (Beijing, China). The NTD of NP consisted of amino acid residues 44–174, and the CTD of NP consisted of amino acid residues 255–364. Both the NTD and CTD of truncated recombinant NP were purchased from ACRO Biosystems (Beijing, China). An extraction buffer containing PBS with 0.1% sodium azide, 0.5% sodium hydroxide, and 1% albumin bovine serum was obtained from Panion & BF Biotech Inc. (Taipei, Taiwan).

### 4.2. Generation and Purification of mAbs

Anti-NP mAbs were generated according to previously described procedures [43,44]. Female 4–6-week-old BALB/c mice were immunized with wild-type recombinant NP-His. After four inoculations with the same concentration of antigen, the splenocytes from immunized mice were harvested and fused with mouse myeloma NS-1 cells. The fused cells were cultured in DMEM supplemented with 15% FBS, HAT medium, and hybridoma cloning factors in 96-well tissue culture plates. Two weeks after fusion, the culture supernatants were screened by ELISA. Selected clones were subcloned via limiting dilutions. Hybridoma clones were isotyped using a commercially available ELISA isotyping kit (Southern Biotech, Birmingham, AL, USA). Ascite fluids were produced in pristine-primed BALB/c mice. mAbs were affinity-purified with standard protein G-Sepharose 4 Fast Flow (GE Healthcare, Chicago, IL, USA) according to the manufacturer’s directions. All animal experiments were approved by the Academia Sinica Institutional Animal Care and Use Committee (IACUC protocol No. 20051468).

### 4.3. Screening of mAbs against NP by ELISA

ELISA was performed as previously described [45]. The wells of 96-well microplates were coated with NP-His protein (0.5 μg/mL). Then, the microplates were washed with PBST (PBS containing 0.1% Tween 20), followed by blocking with 1% bovine serum albumin (BSA). Serial dilutions of the indicated NP antibodies were added to the wells and incubated at room temperature (RT) for 1 h. After washing with PBST, horseradish peroxidase (HRP)-conjugated anti-mouse IgG (Jackson ImmunoResearch Laboratories, Inc., West Grove, PA, USA) was incubated in the wells at RT for 1 h. Finally, the plates were incubated with chromogenic substrate 3,3′,5,5,’-tetramethylbenzidine (TMB; Sigma-Aldrich, St. Louis, MO, USA). The reactions were stopped with 3-N HCl, and the optical density was measured using a microplate reader at 450 nm.

### 4.4. Western Blot Analysis

For the expression of NP in eukaryotic cells, the NP-coding region was cloned into the pcDNA3.1 vector in-frame with a Flag tag. The plasmid DNA pcDNA3.1-NP-Flag was transiently transfected into 293T cells using PolyJet (SignaGen Laboratories, Rockville, MD, USA). After 48 h, the cells were harvested and lysed with RIPA buffer. The cell lysates were separated by SDS-PAGE and transferred to a nitrocellulose membrane (Hybond-C Super). The membranes were blocked with 5% skimmed milk in PBS and incubated with anti-NP mAbs. The blot was then treated with horseradish peroxidase-conjugated goat anti-mouse immunoglobulin (Jackson ImmunoResearch Laboratories, Inc., West Grove, PA, USA) and developed with enhanced chemiluminescence reagents (ECL, Thermo Fisher Scientific, Waltham, MA, USA).

### 4.5. Lateral Flow Immunoassay (LFIA) Strip Preparation

The LFIA strips were manufactured by Panion & BF Biotech Inc. (Taipei, Taiwan). The design of the colorimetric LFIA is a sandwich format in which anti-NP mAbs capture NP in samples. NP-mAb-7 and sheep antibody were labeled with red and black latex microspheres, respectively. Then, the antibodies were sprayed onto the pretreated conjugation pad. NP-mAb-40 was immobilized on the test line, and anti-goat IgG was immobilized on the control line of the nitrocellulose membrane.

In the test procedure, viral NP from a nasopharyngeal swab of a COVID-19 patient was extracted with the extraction buffer, which was then applied to the conjugation pad. The NP antigen was captured by NP-mAb-7 conjugated to red microspheres in an immunocomplex. The immunocomplex could then bind to NP-mAb-40 on the test line, resulting in a red positive signal. The black microsphere-labeled sheep antibody was captured by anti-goat IgG on the control line, producing a black control signal.

The dimensions of the LFIA test strips were 80 × 3.8 mm. Each LFIA strip consisted of a sample pad, conjugate pad, absorbent pad, and nitrocellulose membranes, which were assembled onto a backing card. Both sample pads and conjugate pads were pretreated with a buffer for 6 h, then dried overnight at room temperature and stored at room temperature before use. The sample pad was treated with 100-mM tris buffer containing 0.5% Triton X-100, and the conjugate pad was treated with 100-mM phosphate buffer containing 1% Triton X-100. Using a programmable automatic dispenser, NP-mAb-40 and anti-goat IgG were dispensed onto the nitrocellulose membrane as the test line and control line, respectively. The spacing between the control line and test line was 5 mm. The strips were then dried overnight at 37 °C.

### 4.6. Immunofluorescence Assay (IFA)

NP-expressing Vero E6 cells were grown on coverslips to 80% confluence. Cells were washed twice with PBS and fixed with 4% paraformaldehyde; after which, the cells were permeabilized with 0.1% TritonX-100 and blocked with 3% BSA. The cells were then probed with 1-μg/mL anti-NP mAbs and incubated at 4 °C overnight. After washing with PBS, the cells were stained with FITC-labeled anti-mouse IgG (Jackson ImmunoResearch Laboratories, Inc., West Grove, PA, USA). The nuclei were stained with DAPI. Fluorescence images were captured with an inverted fluorescence microscope (Axiovert 200M; Carl Zeiss AG, Oberkochen, Germany).

### 4.7. Detection of NP from SARS-CoV-2-Infected Cells by NP-mAb LFIA

SARS-CoV-2 (hCoV-19/Taiwan/4/2020, GISAID accession ID: EPI_ISL_411927)-infected Vero E6 cell lysates and cultured supernatants were kindly provided by the laboratory of Dr. Yi-Ling Lin at the Institute of Biomedical Sciences, Academia Sinica. Cells were lysed with RIPA buffer; then, the supernatants were collected after centrifugation. The RIPA lysis buffer was mixed 1:1 with the cell supernatants and incubated for 10 min at room temperature in a P3 laboratory. Each sample was inactivated by gamma-irradiation, and five-fold serial dilutions were made using the extraction buffer. The dilutions were applied at a 100-μL volume to the indicated LFIAs. The diluted sample flowed from the sample pad to the absorbent pad by a capillary action. The present viral NP antigen bound to anti-SARS-CoV-2 NP mAb-conjugated latex on the conjugation pad, and the immunocomplex was captured by the second mAb, resulting in a visually identifiable red color on the test line. The latex-conjugated sheep antibody was captured with anti-goat IgG and formed a black color at the control line. The LFIA procedure could be finished within 10 min.

### 4.8. LFIA limit of Detection (LOD)

A preliminary LOD screening was performed with 2-fold serial dilutions of inactivated SARS-CoV-2 virus and a human nasal matrix for LFIA. Each condition was repeated three times. Based on the results of preliminary LOD screening, the LOD confirmation study was performed with an additional 20 replicates of each point near the LOD of SARS-CoV-2 to find the lowest dilution with a 100% detection rate. The LFIA is described as above.

### 4.9. Cross-Reactivity Assays of Human Respiratory-Associated Pathogens

Inactivated wild-type SARS-CoV-2 was obtained from BEI resources (NR-52287, isolate USA-WA1/2020) and the laboratory of Dr. Yi-Ling Lin at the Institute of Biomedical Sciences, Academia Sinica (hCoV-19/Taiwan/4/2020). Several respiratory-associated viruses obtained from the Taiwan FDA were analyzed to detect the LFIA specificity. The viruses included the influenza A (Brisbane H1N1) and B (Maryland) viruses, parainfluenza virus type 3, respiratory syncytial virus type B, adenovirus type 5, and rhinovirus A40. In addition, a cross-reactivity to human pathogens was determined for the twelve viruses and ten bacteria listed in Supplementary Table S2. The viruses were obtained from zeptometrix and the Research Center for Emerging Viral Infections (RCEVI) of Chang Gung University (CGU), Taiwan, including HCoV-2293, HCoV-OC43, adenovirus type 7, enterovirus types 68 and 71, human parainfluenza virus, influenza A subtypes H1N1 and H3N2, influenza B Victoria and Yamagata lineages, respiratory syncytial virus type B, and rhinovirus. Bacteria were obtained from the Bioresource Collection and Research Center (BCRC) and RCEVI of CGU; the strains included: *Bordetella pertussis*, *Chlamydia pneumoniae*, *Escherichia coli*, *Haemophilus influenzae*, *Mycoplasma pneumoniae*, *Pseudomonas aeruginosa*, *Staphylococcus aureus*, *Staphylococcus epidermidis*, *Streptococcus pneumoniae*, and *Streptococcus pyogenes.* The indicated concentrations of the pathogen were spiked into the extraction buffer. A swab with or without a 2-fold LOD of SARS-CoV-2 was deposited in the extraction buffer container. The LFIA was performed as described above. Each experiment was performed in triplicate.

### 4.10. Cross-Reactivity and Interference Assays

The cross-reactivity of the rapid test for common interfering substances in human nasal isolates was analyzed at the concentrations shown in Supplementary Table S3. The interfering substances included mucin, whole blood, aspirin, dextromethorphan, diphenhydramine HCl, hemoglobin, Hosoon Troches, nasal washing salt, nasal ointment, NASONEX aqueous nasal spray, oxymetazoline HCl, phenylephrine HCl, postan, swinin nasal sprays, and ibuprofen. These interfering substances were obtained from Panion & BF Biotech Inc. (Taipei, Taiwan). The indicated interfering substance was added into the extraction buffer. Swabs with or without a 2-fold LOD of SARS-CoV-2 were deposited into extraction buffer containers, and the procedure was performed. Each experiment was performed in triplicate.

### 4.11. Accelerated Stability Testing

We followed the current Clinical and Laboratory Standards Institute (CLSI) guideline EP25-A to evaluate the stability of LFIA [46]. The variation of the signal intensity between room temperature (30 °C) and high temperature conditions (60 °C) in accelerated stability studies should be within 0.5 grade of the intensity level and last for at least a 16-day interval, which is equal to a 12-month shelf-life for the reagents. These requirements are according to the Standard Guide for Accelerated Aging of Sterile Barrier Systems for Medical Devices, ASTM International, F1980.

### 4.12. Clinical Studies

The clinical trial was an open-label, nonrandomized study with a planned enrollment of 60 subjects over 20 years old who provided a complete informed consent form. The study was approved by the institutional review board (IRB) at Taoyuan General Hospital, Ministry of Health and Welfare, Taiwan (IRB No. TYGH109032). The inclusion criteria required at least one of the following clinical and epidemiological conditions: fever and other respiratory symptoms within seven days of the onset of symptoms and pneumonia confirmed by clinical, radiological, and pathological examination. The epidemiological conditions involved the people who had a contact history with COVID-19 patients through travel, occupation, contact, and cluster (TOCC). A COVID-19 diagnosis was confirmed by a physician with the gold standard method of RT-PCR using a Roche Cobas^®^ 6800 automated system that was granted Emergency Use Authorization by the US FDA. For the SARS-CoV-2 test, 0.6 mL of a nasopharyngeal sample in a transport medium was loaded onto the Roche Cobas^®^ 6800, where it was mixed with master mix reagents consisting of SARS-CoV-2-specific primers and fluorescence-labeled probes targeting the viral nonstructural region of ORF1a/b and a structural protein in a conserved region of the envelope gene. The cycle threshold (Ct) values were reported by the Cobas^®^ SARS-CoV-2 tests. The limit of detection for this test was determined to be 0.007 TCID_50_/mL. All protocols were performed according to the manufacturer’s instructions.

The antigen rapid test was performed by medical personnel (Taoyuan General Hospital, Ministry of Health and Welfare, Taoyuan, Taiwan) using the appropriate standard operating protocols. Briefly, a clinical specimen was collected with a nasopharyngeal swab and deposited with five twisting motions in an extraction buffer container. After 1 min, the swab was removed, and a test trip was loaded directly from the container. The viral antigen presents in the sample bound to specific mouse monoclonal antibodies against SARS-CoV-2 NP, causing a visually identifiable red color to accumulate at the test line within 10 min. Sensitivity, specificity, and accuracy were calculated as recommended by current CLSI guideline EP-12 [47].

## Figures and Tables

**Table 1:** Characterization of the mAb activities against the NP of SARS-CoV-2.

mAbs	Isotype	ELISA*	WB	IFA	Cross-Reactivity with NPs of Human CoV Strains					
					SARS-CoV	MERS-CoV	HCoV-HKU1	HCoV-OC43	HCoV-229E	HCoV-NL63
NP-mAb-1	n.d.	+	+	n.d.	+	−	−	−	−	−
NP-mAb-2	n.d.	+	−	n.d.	+	−	−	−	−	−
NP-mAb-3	n.d.	++	−	n.d.	+	−	−	−	−	−
NP-mAb-4	n.d.	+	+	n.d.	+	−	−	−	−	−
NP-mAb-5	n.d.	++	+	n.d.	−	−	−	−	−	−
NP-mAb-6	n.d.	+	−	n.d.	+	−	−	−	−	−
NP-mAb-7	IgG1, κ	++	+	+	+	−	−	−	−	−
NP-mAb-8	n.d.	+	+	n.d.	+	−	−	−	−	−
NP-mAb-9	n.d.	+	+	n.d.	−	−	−	−	−	−
NP-mAb-11	n.d.	+	−	n.d.	+	−	−	−	−	−
NP-mAb-12	n.d.	+	+	n.d.	−	−	−	−	−	−
NP-mAb-13	n.d.	+	−	n.d.	+	−	−	−	−	−
NP-mAb-14	n.d.	+	−	n.d.	−	−	−	−	−	−
NP-mAb-15	n.d.	+	−	n.d.	−	−	−	−	−	−
NP-mAb-18	n.d.	++	−	n.d.	+	−	−	−	−	−
NP-mAb-19	n.d.	+	−	n.d.	n.d.	n.d.	n.d.	n.d.	n.d.	n.d.
NP-mAb-21	n.d.	+	+	n.d.	−	−	−	−	−	−
NP-mAb-22	n.d.	++	−	n.d.	+	−	−	−	−	−
NP-mAb-23	n.d.	+	+	n.d.	+	−	−	−	−	−
NP-mAb-24	n.d.	++	−	n.d.	+	−	−	−	−	−
NP-mAb-25	n.d.	++	−	n.d.	−	−	−	−	−	−
NP-mAb-26	n.d.	+	+	n.d.	+	−	−	−	−	−
NP-mAb-27	n.d.	+	+	n.d.	+	−	−	−	−	−
NP-mAb-30	n.d.	++	−	n.d.	+	−	−	−	−	−
NP-mAb-33	n.d.	++	−	n.d.	+	−	−	−	−	−
NP-mAb-35	n.d.	+	−	n.d.	−	−	−	−	−	−
NP-mAb-39	IgG1, κ	+++	+	−	−	−	−	−	−	−
NP-mAb-40	IgG1, κ	+++	+	−	+	−	−	−	−	−
NP-mAb-42	IgG1, κ	+++	+	−	−	−	−	−	−	−
NP-mAb-43	n.d.	+	+	n.d.	+	−	−	−	−	−
NP-mAb-46	IgG1, κ	+++	+	−	−	−	−	−	−	−
NP-mAb-49	IgG1, κ	+++	−	−	−	−	−	−	−	−
NP-mAb-50	n.d.	++	−	n.d.	+	−	−	−	−	−
NP-mAb-51	IgG1, κ	++	+	+	−	−	−	−	−	−
NP-mAb-52	IgG1, κ	+++	+	−	−	−	−	−	−	−
NP-mAb-53	IgG1, κ	+++	+	+	+	−	−	−	−	−
NP-mAb-54	n.d.	+	+	n.d.	n.d.	−	−	−	−	−
NP-mAb-55	n.d.	+++	+	n.d.	n.d.	−	−	−	−	−
NP-mAb-56	n.d.	++	+	n.d.	+	−	−	−	−	−
NP-mAb-61	n.d.	++	+	n.d.	n.d.	−	−	−	−	−
NP-mAb-62	n.d.	++	+	n.d.	n.d.	−	−	−	−	−

Note: +, positive results; −, negative results; n.d., not determined; ELISA*: +, weak; ++, medium; +++, strong.

**Table 2:** LOD for NP-mAb-40/7 LFIA strips using inactivated SARS-CoV-2 (USA-WA1/2020) in the human nasal matrix.

**Inactivated SARS-CoV-2 Conc. (TCID_50_/mL)**		**Positive/Total**	**Positive Percentage**
1	2.19 × 10^3^	9/9	100%
2	1.09 × 10^3^	9/9	100%
3	5.47 × 10^2^	9/9	100%
4	2.73 × 10^2^	8/9	89%
**Inactivated SARS-CoV-2 Conc.** **(TCID_50_/mL)**		**Positive/Total**	**Positive Percentage**
5.47 × 10^2^		20/20	100%

**Table 3:** Demographic characteristics and RT-PCR results of the included patients (*n* = 60).

**Gender**	
Male	50%
Female	50%
**Age (Years)**	
Range	16–80
Average	45.3
Median	43
IQR	23.5
**RT-PCR-Positive Samples (*n*)**	10
Ct values	
Range	16–29.5
Average	22.5
Median	21.1

Note: IQR, interquartile range; Ct, cycle threshold.

**Table 4:** Clinical performance of the NP-mAb-40/7 LFIA strips.

RT-PCR		NP-mAb-40/7 LFIA Strips							
		Positive	Negative	Sensitivity		Specificity		Accuracy	
				%	95% CI	%	95% CI	%	95% CI
Positive	10	9	1	90	55.5–99.8	100	92.9–100	98.3	91–100
Negative	50	0	50						

## Data Availability

All data are included within the article and supplementary materials.

## Supplementary Materials

The following are available online at https://www.mdpi.com/article/10.3390/ijms222212412/s1.
