# Supplementary material for: Monoclonal Antibodies against Nucleocapsid Protein of SARS-CoV-2 Variants for Detection of COVID-19
Source: Int J Mol Sci. 2021 Nov 17;22(22):12412. doi: 10.3390/ijms222212412 (PMC8623253; doi:10.3390/ijms222212412)
Supplement: Supplementary file 1 [file ijms-22-12412-s001.zip › ijms-1428677-supplementary.pdf]

International Journal of *Molecular Sciences*

MDPI

Article

Monoclonal Antibodies against Nucleocapsid Protein of SARS-CoV-2 Variants for Detection of COVID-19

Ruei-Min Lu [1,†], Shih-Han Ko [1,†], Wan-Yu Chen [2], Yu-Ling Chang [1], Hsiu-Ting Lin [2] and Han-Chung Wu [1,2,*]

[1] Biomedical Translation Research Center (BioTReC), Academia Sinica, Taipei 11529, Taiwan; reminlu@gate.sinica.edu.tw (R.-M.L.); shko@gate.sinica.edu.tw (S.-H.K.); a29259963@hotmail.com (Y.-L.C.)
[2] Institute of Cellular and Organismic Biology, Academia Sinica, Taipei 11529, Taiwan; sirirem59@gmail.com (W.-Y.C.); 3772.monico@gmail.com (H.-T.L.)
* Correspondence: hcw0928@gate.sinica.edu.tw
† These authors contributed equally.

Supplementary materials

Table S1. Prototype lateral flow immunoassays using different antibody pairs.

**mAb pair**	**Capture mAb**					7									39				
	**Detection mAb**	7	39	40	42	46	49	51	52	53	7	39	40	42	46	49	51	52	53
**Antigen**	**NP from *E.coli***	0.1	0.5	0.5	0	0	0	0	0	0.5	1.5	0	1	0	0	0	0	0	2
	**NP from 293 cells**	1	0	1	0	0	0	0	0.1	1.5	0.5	0	0	0	0	0	0	0	0.5
**Sum of rating**		1.1	0.5	1.5	0	0	0	0	0.1	2	2	0	1	0	0	0	0	0	2.5
**mAb pair**	**Capture mAb**					40									42				
	**Detection mAb**	7	39	40	42	46	49	51	52	53	7	39	40	42	46	49	51	52	53
**Antigen**	**NP from *E. coli***	1	0.5	0	0	0	0	0	0	1	0	0	0	0	0	0	0	0	0
	**NP from 293T cells**	1.5	0	0	0	0	0	0	0	1	0.5	0	0	0	0	0	0	0	0
**Sum of rating**		2.5	0.5	0	0	0	0	0	0	2	0.5	0	0	0	0	0	0	0	0
**mAb pair**	**Capture mAb**					46									49				
	**Detection mAb**	7	39	40	42	46	49	51	52	53	7	39	40	42	46	49	51	52	53
**Antigen**	**NP from *E. coli***	0	0	0.5	0	0	0	0	0	0.5	0.1	0.5	0.5	0	0	0	0	0	0.5
	**NP from 293T cells**	0.5	0	0	0	0	0	0	0	0.1	1	0	0.5	0	0	0	0	0	0.5
**Sum of rating**		0.5	0	0.5	0	0	0	0	0	0.6	1.1	0.5	1	0	0	0	0	0	1
**mAb pair**	**Capture mAb**					51									52				
	**Detection mAb**	7	39	40	42	46	49	51	52	53	7	39	40	42	46	49	51	52	53
**Antigen**	**NP from *E. coli***	0	0	0	0	0	0	0	0	0	0.1	0	0.5	0	0	0	0	0	1.5
	**NP from 293T cells**	0.5	0	0	0	0	0	0	0	0.1	1.5	0	1	0	0	0	0	0	1
**Sum of rating**		0.5	0	0	0	0	0	0	0	0.1	1.6	0	1.5	0	0	0	0	0	2.5
**mAb pair**	**Capture mAb**					53													
	**Detection mAb**	7	39	40	42	46	49	51	52	53									
**Antigen**	**NP from *E. coli***	1	2	0.5	0	0	0	0	0.5	0					-				
	**NP from 293T cells**	2	0	1	0	0	0	0	0	0									
**Sum of rating**		3	2	1.5	0	0	0	0	0.5	0									

Note: One hundred microliters of 100 ng/mL recombinant NP purified from *E.coli* and 293T cells was used for each antigen sample. Rating chart (intensity range from 0 to 5) shows grading of the positive and negative results. Latex color intensity equal or greater than 0.5 was considered a positive specimen (high positive: ≥ 3; medium positive ≥ 2; low positive ≥ 1); less than 0.5 was considered negative. Results were interpreted within 10 min of sample application.

Table S2. The list of human common respiratory pathogens of cross-reactivity study.

**Bacteria Panel**	**Bacteria Counts (CFU/mL)**	**Negative Group (without SARS-CoV-2)**			**Positive Group (with SARS-CoV-2)**		
Bordetella pertussis	1×10^{6}	−	−	−	+	+	+
Chlamydia pneumoniae	1×10^{6}	−	−	−	+	+	+
Escherichia coli	2×10^{8}	−	−	−	+	+	+
Haemophilus influenzae	3×10^{8}	−	−	−	+	+	+
Mycoplasma pneumoniae	1×10^{6}	−	−	−	+	+	+
Pseudomonas aeruginosa	3×10^{8}	−	−	−	+	+	+
Staphylococcus aureus	7×10^{8}	−	−	−	+	+	+
Staphylococcus epidermidis	6×10^{8}	−	−	−	+	+	+
Streptococcus pneumoniae	7×10^{8}	−	−	−	+	+	+
Streptococcus pyogenes	1×10^{6}	−	−	−	+	+	+
**Viral Panel**	**Virus Titer**	**Negative Group (without SARS-CoV-2)**			**Positive Group (with SARS-CoV-2)**		
**Adenovirus type 7**	2.81×10^{5} $TCID_{50}$/mL	−	−	−	+	+	+
**Human coronavirus 229E**	6×10^{5} PFU/mL	−	−	−	+	+	+
**Human coronavirus OC43**	1×10^{5} PFU/mL	−	−	−	+	+	+
**Enterovirus type 68**	8.91×10^{5} $TCID_{50}$/ml	−	−	−	+	+	+
**Enterovirus type 71**	2.11×10^{7} $TCID_{50}$/mL	−	−	−	+	+	+
**Human Parainfluenza Virus (HPIV)**	1.19×10^{5} $TCID_{50}$/mL	−	−	−	+	+	+
**Influenza A -H1N1**	1.7×10^{5} $TCID_{50}$/mL	−	−	−	+	+	+
**Influenza A -H3N2**	1.8×10^{5} $TCID_{50}$/mL	−	−	−	+	+	+
**Influenza B -Vic**	9.2×10^{5} $TCID_{50}$/mL	−	−	−	+	+	+
**Influenza B -Yam**	4×10^{5} $TCID_{50}$/mL	−	−	−	+	+	+
**Respiratory syncytial virus type B**	1×10^{5} PFU/mL	−	−	−	+	+	+
**Rhinovirus**	6.5×10^{6} $TCID_{50}$/mL	−	−	−	+	+	+

Note: CFU: Colony-Forming Units; PFU: Plaque-Forming Units; $TCID_{50}$: 50% of Tissue Culture Infectious Dose; −: Negative result; +: Positive result.

Table S3. Materials used in interference assays.

Interfering Substances	Concentration	Negative Group (without SARS-CoV-2)			Positive Group (with SARS-CoV-2)		
**Mucin**	4%	−	−	−	+	+	+
**Whole blood**	5%	−	−	−	+	+	+
**Aspirin**	20 mg/mL	−	−	−	+	+	+
**Dextromethorphan**	10 mg/mL	−	−	−	+	+	+
**Diphenhydramine HCl**	5 mg/mL	−	−	−	+	+	+
**Hemoglobin**	20 mg/mL	−	−	−	+	+	+
**Hosoon Troches (ROOT)**	20 mg/mL	−	−	−	+	+	+
**Nasal Washing Salt**	20 mg/mL	−	−	−	+	+	+
**Nasal Ointment**	10%	−	−	−	+	+	+
**NASONEX Aqueous Nasal Spray**	10%	−	−	−	+	+	+
**Oxymetazoline HCl**	10 mg/mL	−	−	−	+	+	+
**Phenylephrine HCl**	100 mg/mL	−	−	−	+	+	+
**Postan**	20 mg/mL	−	−	−	+	+	+
**Swinin nasal sprays**	10%	−	−	−	+	+	+
**Ibuprofen**	20 mg/mL	−	−	−	+	+	+

Note: − : Negative result; + : Positive result.

Table S4. Comparison of COVID-19 NP antigen LFIA tests.

Product Name	Company/Sponsor	LoD ($TCID_{50}$/mL)	Accuracy Information	Type of Sample	Approved
**BinaxNOW COVID-19 Ag Card**	Abbott Diagnostics Scarborough, Inc.	140.6	Sensitivity: 84.6%<br>Specificity: 98.5%	NS	US EUA
**SARS-CoV-2 Rapid Antigen Test**	Roche	494	Sensitivity: 95.5%<br>Specificity: 99.2%	NP	CE marking
**NP-mAb-40/7 LFIA (Acadecise)**	Academia Sinica, Taipei, Taiwan	547	Sensitivity: ≥ 99%<br>Specificity: ≥ 99%	NP	TFDA EUA
**Sienna-Clarity COVID-19 Antigen Rapid Test Cassette**	Salofa Oy	1250	Sensitivity: 87.5%<br>Specificity: 98.9%	NP	US EUA
**SPERA COVID-19 Ag Test**	Ttrava Health	1560	Sensitivity: 91.8%<br>Specificity: 96.9%	NS	US EUA
**STANDARD Q COVID-19**	SD-Biosensor	1981	Sensitivity: 96.5%<br>Specificity: 99.7%	NP	CE marking
**InteliSwab COVID-19 Rapid Test Pro**	OraSure Technologies, Inc.	2500	Sensitivity: 84%<br>Specificity: 98%	NS	US EUA

Note: LoD, limit of detection; NS, nasal swab; NP, nasopharyngeal swab; EUA, Emergency Use Authorization; TFDA, Taiwan Food and Drug Administration. Arranged in LoD.

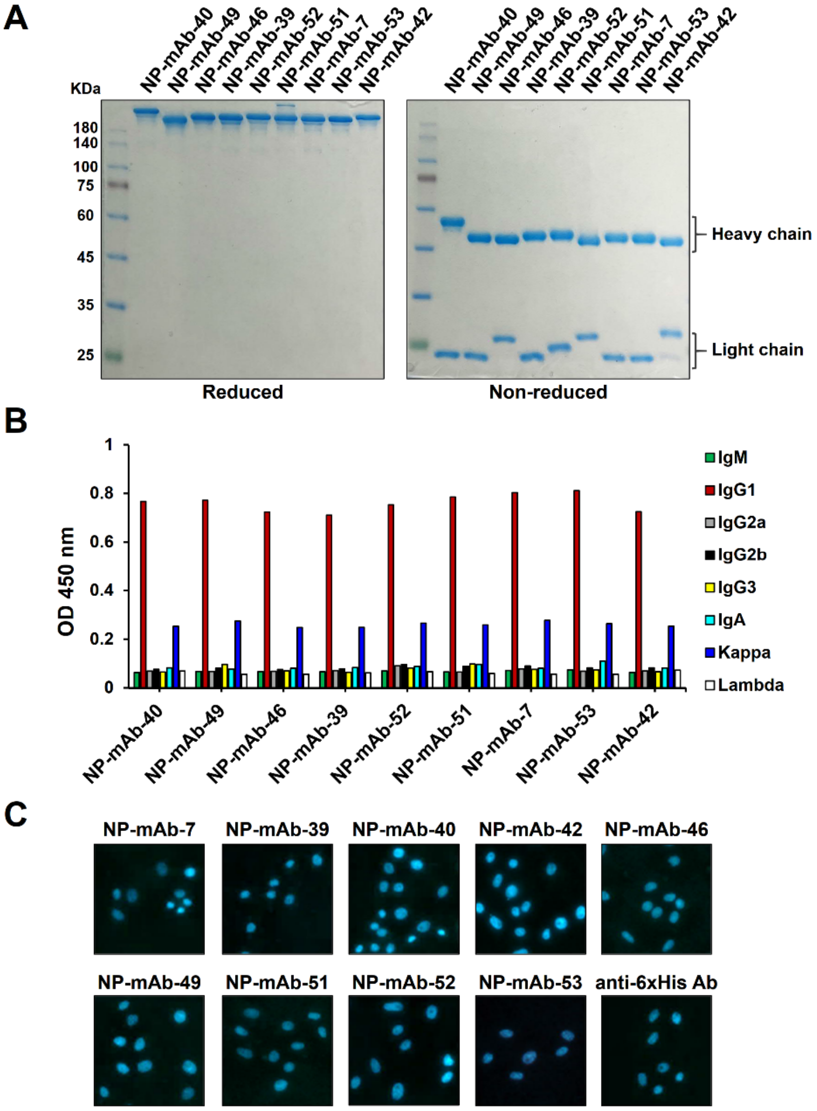

Figure S1. Characterization of NP-specific antibodies. (**A**) Nine antibodies were produced in ascites and purified by protein G sepharose. SDS-PAGE and Coomassie blue staining were conducted to identify heavy chain and light chain of each antibody. (**B**) Nine anti-NP antibodies were isotyped with ELISA-based isotyping kits. (**C**) Immunofluorescence staining corresponds to Figure 1E. Mock Vero cells were probed with 1 μg/mL anti-NP mAb, followed by FITC-goat-anti-mouse IgG. Anti-6xHis Ab with rhodamine-conjugated secondary antibody was used to confirm NP expression.
